# Neuronal Damage in Murine Experimental Cerebral Malaria, Implications for Neuronal Repair and Sequelae

**DOI:** 10.3390/cells14110807

**Published:** 2025-05-30

**Authors:** Monique F. Stins, Irene Gramaglia, Joyce Velez, Carlos A. Pardo, Henri van der Heyde

**Affiliations:** 1 Malaria Research Institute, Johns Hopkins Bloomberg School of Public Health, Baltimore, MD 21205, USA; 2 La Jolla Infectious Diseases Institute, LJIDI, San Diego, CA 92130, USA; 3 Biomedical Research Institute of Southern California, Oceanside, CA 92046, USA; 4 Department of Neurology, Division Neuroimmunology and Neurological Infections, Johns Hopkins School of Medicine, Baltimore, MD 21287, USA

**Keywords:** cerebral malaria, neurons, murine experimental malaria, blood brain barrier, NeuN, neurofilament light chain, DCX, myelin basic protein, neuroprogenitors

## Abstract

Cerebral malaria (CM) is a deadly complication of *P. falciparum* infection. Although adults with CM have a higher mortality rate, CM affects mostly children under the age of 5 years. Neurological symptoms and signs include impaired consciousness, coma, seizures, and increased intracranial hypertension. Upon survival of a CM episode, persistent neurologic deficits occur in a subset of surviving children. These sequelae include recurrent seizures, behavioral deficits, loss of developmental milestones, learning disabilities and attention deficit hyperactivity disorder, which can remain with the survivors. The underlying neuropathology of these post CM neurologic sequelae are unclear. Therefore, we probed the extensive neuronal damage that occurs in an experimental murine model of cerebral malaria (eCM), focusing on the hippocampus. In addition, we explored responses of neuro-progenitor cells (NPC’s) and potential repair mechanisms. We report here that *Plasmodium* infection causes extensive neuronal damage in the hippocampus, characterized by a loss of neuronal NeuN and double cortin (DCX) immunostaining in eCM mice. On day 6 of eCM we also observed increased neurofilament light chain staining, indicative of neuronal fragmentation, which was accompanied by an increase in neurofilament light chain in CSF but not seen in plasma. A concomitant increase in the influx of neuroprogenitor cells in eCM was observed, suggesting ongoing neuronal repair.

## 1. Introduction

CM is one of the serious complications of *P. falciparum* infection. Although adults with CM have a higher mortality rate, CM affects mainly children under the age of five [1,2]. Children who survive CM often remain with neurologic sequela that can be transient and disappear within 3 to 6 months or can persist for years [3]. Sadly, up to 30% of the children who survive CM have increased risks of life-long post-CM neurologic sequelae, including cognitive and behavioral problems [3,4,5,6,7,8,9,10,11]. In particular, neurological deficits can affect developmental milestones, cause attention deficit hyperactivity disorder and reduce quality of life and opportunities [9,12,13,14,15,16]. However, how the *Plasmodium* parasite causes these neurological symptoms is not fully understood.

The hallmark of CM is the sequestration of *Plasmodium*-infected red blood cells in the brain’s vasculature. Interestingly, the intra-erythrocytic *Plasmodium* parasite does not enter the brain by crossing the blood brain barrier (BBB) but still causes brain damage [17]. Several hypotheses have been outlined to explain the neuropathogenesis of CM, such as the mechanical hypothesis, based on the sequestrations of parasitized red blood cells, resulting in hypoperfusion and localized hypoxia [18,19]. Pimonidazole injections identified hypoxia in perivascular neurons, which was confirmed by increased immunoreactivity for hypoxia inducible factor-1 [20]. The hypoxia could contribute to neuronal damage near parasitized micro vessels, as shown in retinal examinations of CM patients [21]. BBB disruption is observed in CM [17,22,23] and neuroimaging in both human CM and in an experimental murine model for CM (eCM) shows evidence of cerebral edema [24,25,26]. The cytokine storm hypothesis [27], postulates that increased peripheral inflammation and circulating chemokines and cytokines significantly contributes to development of CM. Indeed, in addition to peripheral inflammation, high levels of neuroinflammation, as evidenced by the levels of chemokines and cytokines in the cerebrospinal fluid (CSF) [28,29,30,31,32,33], have been suggested to play a key role in CM neuropathogenesis [34]. With a key role for inflammation playing a role in both humans and murine eCM models, the resulting brain swelling can lead ultimately to the loss of life [21,24,25,35]. However, none of these single hypotheses are conclusive and explain the full course of neuronal damage underlying these neurological sequelae and additional host and parasite factors play a role. As we are interested in investigating the pathogenesis of neurological sequelea, we studied characteristics of the neurological damage that may contribute to the neurocognitive deficits in post-CM sequelae, focusing on the hippocampal formation.

The hippocampus, located in the medial temporal lobe, is involved in memory and cognitive function [36,37]. Regions of the hippocampus such as the dentate gyrus (DG) and cornu ammonis (CA) regions, which are central to the cognitive and memory networks, maintain a continuous replenishment of neurons. Hippocampal DG’s newborn neurons are continuously added and develop into granule neurons that progressively extend their axons towards the CA2 and CA3 regions of the hippocampus [38]. Disruption of this process of neurogenesis can lead to seizure activity and cognitive dysfunction [39]. Neuro-inflammation may affect the brain during critical periods of brain development, pathological mechanisms that as occurs in CM, leading to alteration of newborn granule neurons, influencing survival, maturation and replacement of newborn neurons with subsequent long-term neuronal disturbances [40,41]. As memory and learning deficits have been observed in CM survivors, as well in murine experimental CM models [6,9,12,13,14,15,16,42,43], this study focuses on the hippocampal formation neuronal biology.

Neuropathological studies on human CM are limited due to the restricted availability of human CM brain samples. Studies with primate models of CM are very limited due to costs and ethical concerns. The development of experimental murine models of CM, which are low cost and availability of investigational tools, facilitate reliable assessment of the neurobiology of CM. The in vivo murine *Plasmodium berghei*-ANKA (PbA) experimental CM model replicates essential parts of human CM pathology, including the evidence of trapped parasitized red blood cells that disturb blood flow, vascular leakage, astro-neuronal damage, gliosis and impaired neurological dysfunction [42,44,45,46,47,48]. This makes the PbA eCM model a suitable and valuable tool to aid in the study of CM neuropathogenesis. Therefore, we used this widely used murine eCM model to study neuronal damage that may be responsible for the neurological sequelae in CM [42,44,45,47,48,49].

## 2. Materials and Methods

### 2.1. Chemicals and Reagents

Polyvinylpyrrolidone, sodium citrate, sodium tetraborate decahydrate, hydrochloric acid (HCl), ethylene glycol, 4′,6-diamidino-2-phynymindole (DAPI) nucleic acid stain, Triton X-IOO, normal goat serum (NGS), and normal donkey serum (NDS) and a GIEMSA stain kit were purchased from Millipore-Sigma (Burlington, MA, USA). Sodium acetate was purchased from Fisher Scientific (Waltham, MA, USA). Heparin (cat#411210010) was from Acros Organics. The anti-proliferation marker Kiel67 (Ki-67) antibody (1:100) (cat# D3B5) and anti-neurofilament light chain (NFL) antibody (C28E10) (1:200) were purchased from Cell Signaling Technology (Danvers, MA, USA). Anti-bromodeoxyuridine (BrdU) antibody (1:1000) (cat # OOBT0030) was from BioRad/AbD Serotec (Hercules, CA, USA). Anti-doublecortin (DCX) antibody (1:250) (cat# sc-8066) was obtained from Santa Cruz Biotechnology (Dallas, TX, USA). Anti neuron-specific nuclear binding protein (NeuN) antibody (1:250) (cat# MAB3773) was from Millipore (Burlington, MA, USA). Anti-brain myelin protein (BMP) (cat # NB600-71755) antibody (1:200) was from Novus Biotechnologies. ELISA for neurofilament light chain (NFL) was purchased from MyBioSource (San Diego, CA, USA). Secondary antibodies: goat anti-rabbit Alexa fluor (AL) 488, donkey anti-mouse AL555, and donkey anti-goat AL633, donkey anti-mouse lgGIgG-AL555 antibody, goat anti-rat IgG-AL488 antibody, and goat anti-donkey IgG-AL633 were purchased from Life Technologies (Thermofisher, Carlsbad, CA, USA) and used at 1: 250. Prolong gold mounting media (Invitrogen cat#P36935). Phosphate Phosphate-buffered saline was purchased from Corning (Corning, NY, USA).

### 2.2. Animals

To induce experimental cerebral malaria (eCM), six to 10-week-old wild type C57 black mice (C57/Bl6) of both sexes were infected with *Plasmodium berghei* ANKA (PbA) (MR4-BEI resources, Manassas, VA, USA) at 10^6^ PRBCs, intraperitoneal, as previously published [50,51]. The Institutional Animal Care and Use Committee (IACUC) of La Jolla Infectious Disease Institute approved all protocols and procedures. (#LJ14-01 3/7/2014). The mice were maintained on standard chow in standard ventilated cages with enrichment recommended and approved by IACUC. Four independent experiments with each 3–4 mice per group were done. In each experiment the development of disease was assessed daily by measuring the following clinical parameters: (1) righting and gripping reflexes (on a score of 0–5, with 5 exhibiting no impairment), (2) weight, (3) skin temperature, and (4) parasitemia, as previously published [52,53]. Parasitemia was assessed on thin blood films starting on day 4 and every 2 days after, until eCM or up to day 12 for mice that did not progress. Usually, 75–80% of mice would progress to full eCM by day 6. When eCM/neurological signs developed, mice were closely watched and euthanized before they became moribund (eCM score < 4). Only mice that had qualifying combined eCM scores included in this study. To follow neuroprogenitor cells, mice were injected with BrdU (10 mg/200 µL) 3 h before euthanasia, as described by Lee et al. [54]. Avertin (tribromoethanol) anesthetized mice were trans-cardially perfused by gravity with ~15 mL of phosphate buffered saline (PBS) containing 0.1 mg/mL heparin at 4 °C until clear fluid exited, followed by ~20 mL of freshly prepared 4% paraformaldehyde (PFA)/PBS at 4 °C. Whole brains were dissected and post-fixed in 20 mL 4% PFA overnight at 4 °C. Subsequently brains were cryoprotected in solutions with increasing sucrose concentration (15% to 30% sucrose/PBS) and kept at 4 °C. Brains were snap frozen in a clean 5 mL Eppendorf tube by immersion of the tube in 2-Methylbutane with dry ice and stored at −80 °C.

Blood was collected by retro-orbital bleed, as previously described [51], allowed to clot at RT for 30 min and centrifuged at 1500× *g* for 10 min in a refrigerated centrifuge at 4 °C. Serum was then aspirated and placed into fresh tube and frozen at −80 °C until analyzed. Cerebrospinal fluid (CSF) samples were obtained as a terminal procedure by cisternal puncture through the foramen magnum. A small hole was created in the membrane and using a pulled glass hematocrit capillary with a very fine point. CSF was withdrawn through, as described [55]. Approximately 5–10 µL was obtained per mouse diluted in PBS and stored at −80 °C till analysis. Neurofilament light chain (NFL) was determined by ELISA in triplicate.

### 2.3. Preparation of Floating Brain Sections

Mouse brains that had been previously fixed with paraformaldehyde (PFA) and cryopreserved with sucrose were cut into ~40 µm sections using a sliding microtome (Microm HM440 E). Sections were collected in 48-well plates containing 500 µL section storage solution (1% polyvinylpyrrolidone, 40% ethylene glycol, 1M sodium acetate, at pH 6.5). Brain sections from the lateral ventricles and the hippocampus were used for immune-fluorescence incubations. Matching regions of interest for both control and PbA infected brains were selected with the microscope. Per condition, a minimum of 3 mice, with 3 to 4 sections per brain were evaluated. Prior to immunostaining, the sections were washed three times in PBS for 10 min and a fourth wash overnight. Sections probed for BrdU were pre-incubated in 1M HCl for 30 min at 37° C, followed by a 30-min incubation in borate buffer (0.1M sodium tetraborate decahydrate pH 8.5). Sections probed for the nuclear proliferation marker Ki-67 were pre-incubated in 10 mM sodium citrate, 0.05% Triton X-100, 6.0 pH that had been heated to 95 °C and allowed to cool at room temperature, together with the sections for a period of 45 min. All sections were then washed with PBS and blocked in blocking buffer (PBS 0.2% Triton X-100 with 5% normal donkey serum (NDS)), followed by an incubation with primary antibodies diluted in PBS-T 0.1% with 1% NDS. Sections were incubated at 4 °C with gentle rotational rocking for 48 h, washed three times with PBS and incubated in secondary antibodies diluted in PBS-T 0.1% with 1% NDS at 4 °C with gentle rocking for 24 h. During secondary antibody incubation, and in all subsequent steps, sections were shielded from light using aluminum foil to preserve fluorescence. Sections were then washed with PBS and incubated in 300 nM DAPI for 30 min followed by another wash in PBS and mounted onto slides with Prolong gold mounting media. The mounted slides were allowed to dry and viewed using a Zeiss Axioimager M2. Pictures of the sections were taken at same settings for all conditions and processed using Volocity image processing software, version 6.3 (Quorum Technologies). Alternatively, sections were scanned using a Panoramic P1000 scanner (courtesy of Rick Wolfe, Epredia) and viewed with Case viewer software. The background was subtracted with similar settings for all conditions and further cropped and assembled using Microsoft Power-Point.

### 2.4. Data Analysis

Immunofluorescence of brain sections was quantified using Image J/FIJI software (https://imagej.net/software/fiji/, accessed on 26 May 2025) [56]. A manual free shape was drawn around the area of interest (DG and CA regions) for each fluorescent channel and the integrated density was measured. The fluorescence intensity was normalized to the selected area to obtain the mean pixel density per area. Results were reported as percentage control to make comparisons between separate experiments. GraphPad Prism 6.0 software (GraphPad Software; San Diego, CA, USA) and MS Excell were used for statistical analysis. Standard statistical procedures were followed and unless otherwise stated, the data were expressed as mean ± standard error of the mean (SEM). Total Ki67(+) or BrdU + cell numbers were manually counted and analyzed for statistical significance by Student’s *t*-test.

## 3. Results

### 3.1. Neuronal Damage and Altered Morphology in eCM

**Figure 1:**
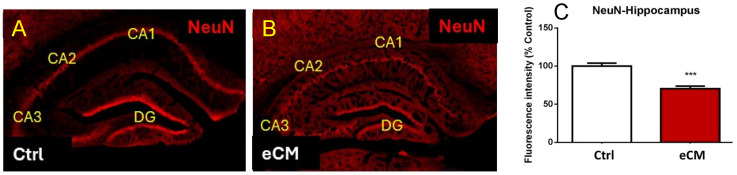
Neuronal damage in eCM. Mature neurons in hippocampus were labeled with anti NeuN-antibody in free floating sections of both control and PbA infected eCM brains. Sections were scanned and post magnification was 5X. (**A**) Control hippocampal neurons showed their characteristic hippocampal architectural pattern. (**B**) At the onset of eCM loss of neuronal anti-NeuN immune staining (red) was observed. (**C**) Quantification of a representative experiment (DG and CA areas) showing decreased NeuN staining over the time course of eCM. (Mean ± SEM graphed, Control n = 6, eCM n = 5, *** *p* < 0.05).

During the course of the PbA infection, parasitemia increased to 8.2 ± 1.03% on day 6. At that time the eCM signs were apparent with eCM clinical scores between 4 and 6 were included, similar to our previous publications [50,51,52,57]. At that time the mice were euthanized, brains dissected and compared with non-infected control mice. Changes in immunostaining for neurons, neuronal progenitors and myelin were assessed in floating sliding sections, as described in the material and methods section. Mature neurons were visualized with anti-NeuN immunostaining, and in the control non-infected mice this revealed the characteristic hippocampal architecture and clear NeuN immuno staining (Figure 1). PbA infection resulted in eCM and a decrease in anti-NeuN immune staining of the hippocampus, indicating a loss of neurons in both DG and CA hippocampal formations.

**Figure 2:**
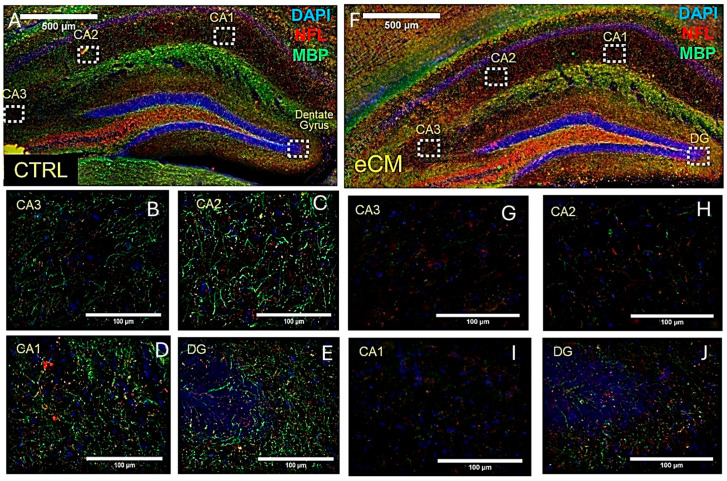
Visualization of neurofilament and myelin damage in eCM. Overview of immunolabeling for neurofilament light chain (NFL) and myelin basic protein (MBP), in a control non-infected mouse (Ctrl) at day 0 and at eCM (bar (**A**,**B**) = 500 µM and bar (**B**–**E**), (**G**–**J**) = 100 µM). (**A**): overview of Day 0 (left panel, (**A**)) shows MBP (green), NFL (red) staining and DAPI (blue). Right panel (**F**,**J**) shows a section from eCM mice. Compared with the control, MBP staining decreased and NFL staining increased in eCM. The bar is 500 microns. Bottom panels: higher magnification and details of indicated areas of the upper figures ((**B**–**E**) = control) and ((**G**–**J**) = eCM). The bar is 100 microns.

As we observed a loss in NeuN neuronal immunostaining, we tested if there would be additional axonal damage by immunolabeling for both myelin basic protein (MBP) and neurofilament light chain (NFL) (Figure 2). This showed an increase in NFL (red) immunostaining and decreased MBP (green) immune staining in eCM, suggesting increased neurofilament breakdown and loss of myelin in eCM brains compared to the control non-infected mice. Especially in the CA1, CA2 and DG areas of the hippocampus, a striking reduction in MBP staining was observed (*p* < 0.05) (Figure 2G–J). These neuronal changes in both NeuN and MBP staining did not appear to be specific to the hippocampus, as the cortical areas were also affected, revealing decreased and ‘interrupted’ MBP immunostaining in eCM.

### 3.2. Increased NFL and Decreased MBP Immunostaining in the Hippocampus in eCM

**Figure 3:**
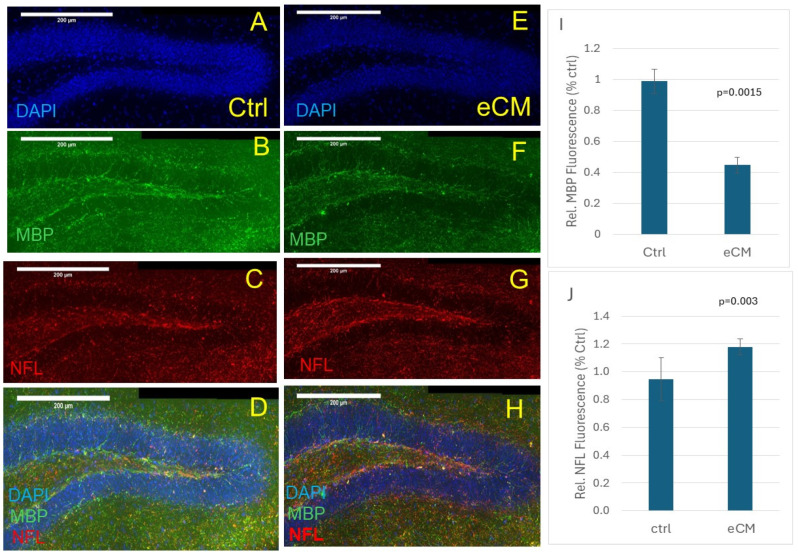
Neuronal damage in the hippocampal DG area. Showing increased NFL (red) immunostaining (**C**,**G**) as well as decreased MBP (green) (**B**,**F**) immunostaining at eCM (**E**–**H**) compared to control (**A**–**D**). Merged pictures shown in (**D**,**H**). Quantification of immunostaining in the DG center for MBP ((**I**), *p* = 0.0015) and NFL ((**J**), *p* = 0.003) for eCM compared to control (n = 3 per group). Mean ± SEM, Bar is 200 microns.

Further focusing on the DG area of the hippocampus, anti-MBP immunostaining (green) was clearly present in the control non-infected mice (Figure 3B). Also here, immunostaining for MBP showed a significant loss of myelin in eCM brains compared to the controls (Figure 3F) (*p* = 0.0015), confirming the above findings. As we observed the loss of total anti-NeuN immune staining of the neurons, we also assessed the appearance of axonal breakdown products, such as fragments of the neurofilament light chain (NFL) (Figure 3). A clear increase in red NFL immunostaining was seen in the eCM sections (Figure 3G) (*p* = 0.003), especially in the DG area, confirming significant axonal damage.

**Figure 4:**
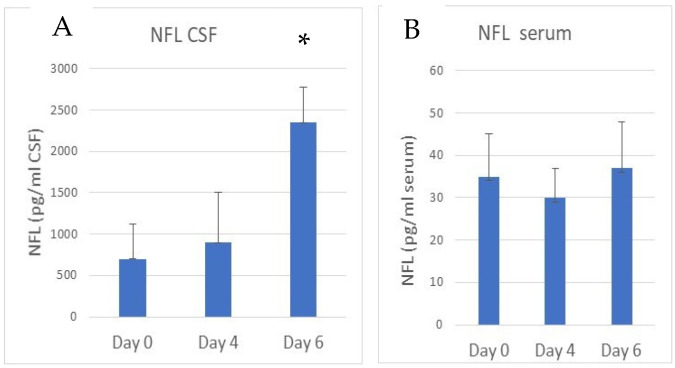
Quantification of appearance of neurofilament light chain in the CSF during eCM. (**A**) Time course of increased amount of neurofilament light chain (NFL) in CSF of PbA infected mice on day 4 and 6, compared to control non-infected mice at day 0. (n = 4 per group) (* *p* < 0.05 compared day 6 to day 0). (**B**) NFL concentration in serum of PbA infected mice on day 4 and 6 compared to day 0. Note the scale difference with high amount of NFL in CSF at day 6 but consistently low amounts of NFL in serum.

Concomitantly with the decline of NeuN immunostaining, the appearance of neurofilament light chain (NFL) fragments can be followed in cerebrospinal fluid (CSF) (Figure 4A). As determined by ELISA, about a 4-fold increase in the presence of NFL was observed in the CSF of the eCM mice on day 6 compared to the control mice (*p* < 0.05). The concentration of NFL in serum was much lower than in the CSF, and no significant difference in serum NFL concentrations were observed in eCM compared to non-infected control mice (Figure 4B).

These results confirmed that neuronal damage occurs in eCM, as evidenced by the decrease in NeuN neuronal staining and the appearance of breakdown products in the form of NFL both in the brain tissue and in the CSF.

### 3.3. Decrease in Immature Neurons and Increased Neuroprogenitors in the Hippocampus During eCM

**Figure 5:**
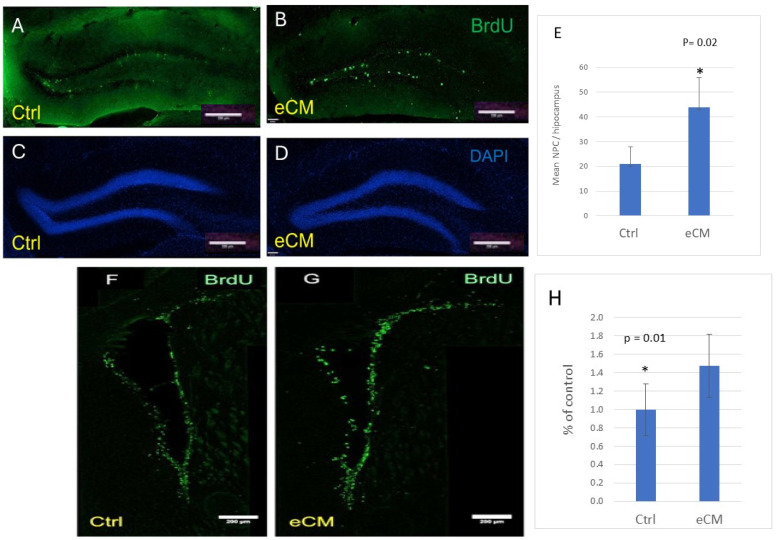
Increase in neuroprogenitors in eCM. Increased numbers of neuroprogenitors were observed in eCM. (**A**,**B**) Presence of proliferating progenitor cells were assessed by BrdU positive cells (green) in the dentate gyrus. (**C**,**D**) Nuclear DAPI staining (blue), (**E**) quantification of BrdU-positive progenitors in eCM shows an increase in BrdU+ cell numbers (* *p* = 0.02). (**F**,**G**) Increase in the number of BrdU+ neuroprogenitors and their staining intensity around the lateral ventricle in eCM compared to control noninfected mice (* *p* = 0.01). (**E**,**H**) Mean ± SD in eCM compared to Ctrl is shown. Bar = 200 µm.

**Figure 6:**
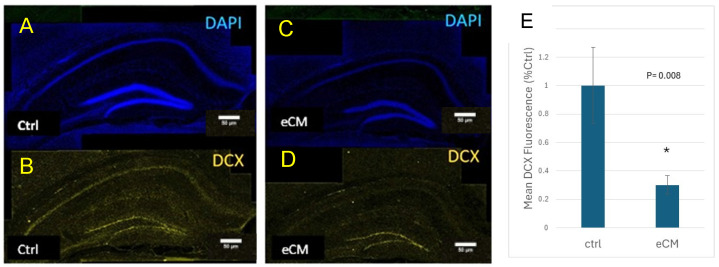
Decreased presence of immature DCX+ progenitors in eCM. To assess the fate of immature neurons during eCM, free floating sections of both control and the PbA infected eCM brains were labeled with anti DCX antibody. (**A**,**B**) Control hippocampus sections revealed DCX immune staining (yellow) in the hippocampus. (**C**,**D**) Sections of eCM brains showed loss of DCX staining in the DG area but a more pronounced loss of expression was observed in the CA areas. (**E**) Quantification shows decreased DCX staining in the DG area of control and eCM. Nuclear staining by DAPI is in blue DCX in yellow/orange. Mean + SEM is shown. (* *p* = 0.008) (n = 3 per group). Bar = 50 µm.

As neuronal immunostaining decreased during the course of eCM, we assessed whether neuronal repair would occur and if neuroprogenitor cells would migrate to areas of neuronal damage. Proliferating neuroprogenitor cells were identified both by BrdU incorporation or presence of the nuclear cell cycle regulating protein Ki67. Neuroprogenitors reside along the lining of the lateral ventricles [17]. In control mice, few BrdU- or Ki67-positive cells were present along the ventricles and in the DG of the hippocampus of control non-infected mice (Figure 5A). However, their numbers significantly increased with PbA infection and eCM development (*p* = 0.02) (Figure 6B). A large increase in their numbers was seen along the ventricle lining and in the DG areas in eCM (Figure 5G). This indicates that there is proliferation of neuroprogenitors and a resulting influx of NPCs into the hippocampus DG area, implicating that neuronal repair is attempted during eCM.

The numbers of immature neurons, as assessed by anti-DCX immune staining, decreased in eCM compared to controls (Figure 6), indicating that immature neurons in both the CA and DG areas of the hippocampus were affected in eCM. These results indicate that in PbA infected mice that develop eCM an increasing amount of neuronal damage occurs, involving both mature neurons, as shown above but also immature neurons.

## 4. Discussion

Using an experimental murine model for CM to further characterize the underlying pathogenic processes we found that extensive neurological damage occurs during eCM, which is in agreement with others [47]. As we were interested in cognitive sequelae, we focused on the hippocampal formation, one of the areas involved in memory and cognition. In the hippocampus, a loss of neurons and myelin was observed, as evidenced by the decreased NeuN- and MBP- immunostaining. Neuronal damage has also been documented in human CM [17,58]. Murine eCM neuropathogenesis also shows evidence of neuronal damage, including increased β-APP staining and tau [45,47]. In contrast, DeSousa et al. [46] did not report any glial-neuronal damage in PbA-infected mice as assessed by FluoJade and anti Iba-1 staining, although they found altered biochemistry and evidence of oxidative damage.

The loss of NeuN immunostaining in the mice in our study was accompanied by a significant increase in NFL fragments. NFL is a marker of axonal damage [59]. In the brains of eCM mice the NFL fragments were released into the CSF and values of up to ~2250 pg/mL, were observed, up from a baseline level of about 700 pg/mL NFL, as determined by ELISA. However, we did not observe a change in serum NFL concentrations in eCM compared to control mice, which was low (~35 pg/mL). Wai et. al. [60] reported neurofilament loss in axons and an increase in NFL plasma values of 317–528 pg/mL in eCM, depending on the level of edema compared to values of ~103 pg/mL in control mice. MRI imaging and electron microscopy of the olfactory bulb, cortex and brainstem also showed a loss of axonal structures, decrease in density and rearrangements of neurofilament in eCM. However, this group did not find a clear correlation of NFL levels with the outcome of the eCM, but did establish that some regeneration of the filaments in the axons occurred. Our immunofluorescent studies with NeuN in the hippocampus also showed a loss of axo-neuronal integrity and appearance of NFL fragments. Our values of NFL in eCM are higher than those of Wai et al. [60], but this can be due to the different methodologies used, such as, intra venous injection of sporozoites in the Wai study versus intra peritoneal injection of PbA infected red blood cells, as in this study. This shows that *Plasmodium* infection has an overall effect on the brain. The fact that we only saw an increase in NFL concentration in the CSF and not in the serum NFL values, could have several underlying causes. As intravenous injection of sporozoites [60] would involve the liver stage, this may lead to differences in timeline and severity of eCM between these two approaches. Like in human CM [21,25,35] there is also edema formation in eCM [24], therefore, there could be a differential dysfunction of the BBB and/or glymphatic drainage into the peripheral circulation. This may affect the appearance of peripherally circulating NFL in eCM, at this stage. However, to clarify this aspect additional future studies would be needed.

The observations on neuronal damage in eCM agree with previously published data on human CM [17,58]. There are limited studies on NFL in CSF and serum in CM, but recently, Balanza et.al [61] reported an in increase in NFL in plasma of African children with uncomplicated malaria and severe malaria. This group reported that NFL levels were higher in more severe cases and when the patients showed neurological manifestations. Datta et al. [62] 2023 also reported an increase in circulating NFL levels in pediatric patients with CM who died, but no CSF levels were reported. Interestingly, they found that elevated circulating NFL levels in patients were associated with worse attention and cognitive testing in children > 5 yrs [63].

In addition to the decrease in NFL, additional axonal damage was found as immunostaining for MBP was significantly decreased. This indicates a loss of myelin around the axons, affecting neuronal transmission. This was apparent in the hippocampal formation, where all areas (CA1-3 and DG) were affected. In addition, we found loss of immunostaining for MBP in other areas of the brain, such as the anterior Cingulate area and secondary motor areas. While assessing the effect on iron on the course and severity of eCM, Leitner et al. [64] also reported loss of MBP in mouse brain fractions. This group postulated that iron deficiency, either by diet or due to infection could lead to more severe myelin damage. Histological analysis in hematoxylin-eosin stain eCM brain sections showed myelin pallor and fragmentation adjacent to congested capillaries [20]. Subsequent ultrastructural studies by this group showed a pathological changes and myelin abnormalities in the corpus callosum in eCM [65], suggesting vulnerability of the oligodendrocytes in eCM and possibly CM as well. Our studies showed a loss of myelin by MBP immune staining in the hippocampus and cortical brain areas. Due to the cellular organization in the hippocampus the neuronal damage, e.g., decreased NeuN and increased NFL immunostaining, appeared more striking in the hippocampal area than in the cortical areas. Neuronal damage in these areas could be similar, but on the other hand, given the inflammatory brain environment in eCM, it is possible that the hippocampal neurons are more sensitive to inflammation and changes in their environment than cortical neurons. In fact, Noel et al. [66] showed that hippocampal neurons were indeed more susceptible to caspases than striatal neurons [66]. Which is of relevance as caspases are also increased in CM [67]. In addition, as *Plasmodium* infection remains intravascular and the parasite does not cross the BBB, it is possible that vascular heterogeneity could play a role as well. Intravascular sequestration and circulating parasite and host factors may have a differential effect on different parts of the vasculature. Hippocampal microvessels may respond differentially in eCM than, for example, vessels in the corpus callosum or cortex. In addition, adjacent hippocampal neurons could be more sensitive to vascular inflammation than cortical neurons. Especially as we are recognizing more and more that vascular heterogeneity may play a role in diverse neurological diseases [68], hippocampal versus cortical brain areas may respond differently in eCM and/or other infections.

The underlying causes for this type of neuronal damage are unclear, but could involve the increased presence of metalloproteases, calpain and/or caspases, as shown in both eCM and CM [67,69]. In addition, the influx of immune cells, such as pathogenic CD8 T cells, which is observed both in murine CM and human CM, can contribute to this [49,70]. Moreover, interactions of PRBC’s, circulating parasite factors and elevated coagulation factors with the endothelium of the BBB causes the brain vascular inflammation leading to a shift in release of chemokines and growth factors from the BBB into the brain, and thus directly or indirectly contributing to the observed neuronal damage [71,72,73,74].

Under normal healthy conditions, neuronal differentiation and growth occurs, especially in the young and growing brain. Neuroprogenitors present in the brain receive cues from their immediate environment to differentiate into the cellular type needed. There are at least two different types of progenitor cells present in the hippocampus: neuronal progenitor cells and oligodendrocyte progenitor cells (see Bonaguidi et al. for a review [75]). However, the identity, fate and functions of these different progenitor cells may overlap during pathological processes. In addition, these different types of progenitors may have a different sensitivity in inflammatory environments. Differential sensitivity of immature versus mature neurons to toxins has been shown in vitro [76]. It is thought that DCX+ progenitors contribute to the generation of oligodendrocytes [77]. The neuro-progenitors from the subventricular zones and DG are thought to differentiate into neurons [77,78].

In the eCM model we see that the immature DCX-positive neurons disappear, possibly because these cells are very sensitive to the highly inflammatory environment in the eCM brain. DCX-positive neurons are also disappearing in other neurological conditions, including multiple sclerosis and cognitive impairments. De Miranda [79] also reported a decrease in DCX positive cells in eCM, concomitant with an increase in inflammatory factors and decreased growth factors, such as brain derived neurotrophic factor and nerve growth factor. In eCM, this could result in the observed myelin damage and reduced MBP immunostaining. At the same time as the DCX+ neurons are disappearing, a large influx of the Ki67/BrdU + progenitors are seen. These cells are located along the lining of the ventricles, along the brain vasculature and in the hippocampus as well [80]. Although we found evidence of neuronal damage in eCM, simultaneous aspects of neuronal repair are observed in eCM. The neuroprogenitors are activated by inflammatory environments, as is the case in the eCM brain [81]. Although the inflammatory eCM environment can trigger progenitor recruitment to sites of injury, expansion of the numbers and differentiation of the progenitors, excessive inflammation can also deregulate the differentiation process and neuronal renewal. Likely, these cells are recruited to the DG in order to repair the neuronal damage in eCM. Depending on whether these newly arrived progenitors receive the proper differentiation cues, there may be a full repair or recovery or with partial repair, cognitive neurologic sequelae may remain.

## 5. Conclusions

CM episodes can result in severe neurologic damage leading to cognitive and behavioral sequelae. In eCM we observed overall neurological damage, such as a loss of NeuN, BMP and increased NFL immune staining. We focused on the hippocampal formation, where we observed a profound decrease in neuronal staining and decreased myelin. In eCM, the brain responded with proliferation and recruitment of neuroprogenitor cells. Further research is needed into the mechanisms of hippocampal injury and repair in CM and the murine eCM model can be used as a reliable model for such studies. In CM patients, post CM-episode follow-up and testing indicated a spectrum of both short and long-term effects [6,82,83]. As certain sequelae are short term, mechanisms to repair the neuronal damage must be in place and the various neuroprogenitor cells can perform these functions. How these processes are exactly regulated and how to steer the neuronal repair process into the direction of full recovery is yet unclear and should be included in the focus of future research efforts. This may not only benefit CM patients but also those that recover from other brain infections and conditions.

## Data Availability

The original contributions presented in this study are included in the article. Further inquiries can be directed to the corresponding author.

## Abbreviations

The following abbreviations are used in this manuscript:

eCM	experimental Cerebral Malaria
CSF	cerebrospinal fluid
DAPI	4′,6-diamidino-2-phenylindole
DCX	Double Cortin
DG	Dentate Gyrus
IACUC	Institutional Animal Care Committee
NFL	neurofilament fragments
PbA	*Plasmodium Berghei* ANKA
PBS	Phosphate buffered saline
PFA	paraformaldehyde
PRBC	*Plasmodium* infected red blood cells
